# PEP-SiteFinder: a tool for the blind identification of peptide binding sites on protein surfaces

**DOI:** 10.1093/nar/gku404

**Published:** 2014-05-06

**Authors:** Adrien Saladin, Julien Rey, Pierre Thévenet, Martin Zacharias, Gautier Moroy, Pierre Tufféry

**Affiliations:** 1 INSERM U973, MTi, F-75205 Paris, France; 2 Université Paris Diderot, Sorbonne Paris Cité, F-75205 Paris, France; 3 Ressource Parisienne en Bioinformatique Structurale, F-75205 Paris, France; 4 Technische Universität München 80333 München, Germany

## Abstract

Peptide–protein interactions are important to many processes of life, particularly for signal transmission or regulatory mechanisms. When no information is known about the interaction between a protein and a peptide, it is of interest to propose candidate sites of interaction at the protein surface, to assist the design of biological experiments to probe the interaction, or to serve as a starting point for more focused *in silico* approaches. PEP-SiteFinder is a tool that will, given the structure of a protein and the sequence of a peptide, identify protein residues predicted to be at peptide–protein interface. PEP-SiteFinder relies on the 3D *de novo* generation of peptide conformations given its sequence. These conformations then undergo a fast blind rigid docking on the complete protein surface, and we have found, as the result of a benchmark over 41 complexes, that the best poses overlap to some extent the experimental patch of interaction for close to 90% complexes. In addition, PEP-SiteFinder also returns a propensity index we have found informative about the confidence of the prediction. The PEP-SiteFinder web server is available at http://bioserv.rpbs.univ-paris-diderot.fr/PEP-SiteFinder.

## INTRODUCTION

Peptide–protein interactions are natural events of life, involving several well-known peptide categories such as hormones, peptides of the central nervous system (1), venom peptides (2), to cite some. In the recent years, peptide–protein interactions have also found an interest in studies targeting protein–protein interactions. For instance, protein–protein interactions can be mediated by short linear peptides that are present in disordered regions of proteins partners (3). There is also a large interest in the design of peptides extracted from structures to mimic protein epitopes in a therapeutic perspective (4), or to design peptide ligands from protein–protein complexes (5). In a general manner, peptides have, in the recent years, had a renewed interest as candidate therapeutics (6,7).

Present *in silico* approaches to assist the functional characterization of peptide–protein interactions can however be largely improved (5,8). Several docking approaches have been developed to predict how a peptide and a protein interact. However, for a majority of these methods, such as DynaDock (9), Rosetta FlexPepDock refinement (10), Rosetta FlexPepDock *ab initio* (11), or PepCrawler (12), the optimization of peptide conformation is only performed in the known binding site. Even the recent HADDOCK peptide docking protocol (13) also requires, to be successful, that the initial position of the peptide is within 5 Å from the peptide in the crystal structures of the complexes. Finally and noteworthy, probably due to large computational costs, only two web servers are currently available for local refinement of a peptide docked into the binding site: FlexPepDock (14) and PepCrawler (12). 

When the binding site is not known, a search on the whole protein surface—global docking or blind docking—must be performed. A classical docking program like AutoDock, designed for the small molecules docking, has been shown efficient for short peptides, such as four residues (15) or seven residues (16). For longer peptides, specific approaches have been developed. Dagliyan *et al*. (17) have shown the relevance of replica exchange all-atom discrete molecular dynamics simulations to identify correctly the peptide-binding sites. Verschueren *et al*. have proposed a protocol to generate models of a peptide at the protein surface, using backbone fragments from the BriX database (18,19). This method has been applied, successfully in most cases, on a dataset of 11 unbound complexes involving peptides of size up to 13 amino acids, and a dataset of 26 bound tetrapeptide-PDZ complexes. Although these programs have demonstrated their ability to carry out blind docking for short peptides, to the best of our knowledge, no web server is currently available.

Instead of performing peptide docking, PepSite (20) aims at predicting the binding site for peptide on the whole protein surface, without returning a complete peptide structure. It is based on spatial position specific scoring matrices (S-PSSMs) for each of the 20 standard residues and three phosphorylated variants. The predicted binding sites for each amino acid are combined with the distance constraints according to the peptide sequence to identify potential binding site for the complete peptide. An online tool is available for an updated version of PepSite (21). It accepts peptides with a maximal size of 10 amino acids. Very recently, PeptiMap, an approach adapting a small molecule hot spot identification protocol to the identification of peptide binding site has been proposed (22). It has been calibrated on a subset of 21 peptide–protein complexes from PeptiDB and validated using a set of nine complexes. It was possible to identify the binding site for 19 and seven of these 21 and nine cases, respectively. It is so far not available online.

Here, we present PEP-SiteFinder, a new tool to identify the peptide-binding site without any knowledge of the potential interaction site. PEP-SiteFinder combines the 3D *de novo* prediction of the peptide structure and the blind docking of peptide predicted conformations using a coarse grained representation. It accepts peptides from four to 36 amino acids. We assess its performance on a third party collection of peptide–protein complexes using the conformation of the unbound protein. We show that PEP-SiteFinder is able to identify relevant information even in cases undergoing conformational changes upon peptide binding. Unlike previous tools, PEP-SiteFinder also quantifies the propensity of protein residues to be at the peptide interface, which we find to correlate with the experimental observations.

## MATERIALS AND METHODS

### Dataset

To benchmark the performance of PEP-SiteFinder, we have used the PeptiDB dataset (23). PeptiDB consists in 103 high-resolution peptide–protein complexes (holo conformation), resolved using X-ray diffraction, with a resolution lower than 2 Å and presenting no sequence identity between two protein monomers more than 70%. The bound peptides have a size between five and 15 amino acids. The protein uncomplexed (apo) conformation is available for 78 complexes and PeptiDB defines a core set of 41 non-redundant complexes, an additional set of 26 complexes structurally redundant with the core set according to Class-Architecture-Topology-Homologous superfamily (CATH) structural classification (24), and a subset of 11 complexes for which large conformational changes occur. Details about the dataset are provided in the Supplementary data. We have performed our tests using the apo conformations and the peptide sequences as input of PEP-SiteFinder and PepSite, and compared the residues predicted in interaction with those at peptide–protein interface in the complexes.

### Protein–peptide interactions

**Figure 1.**
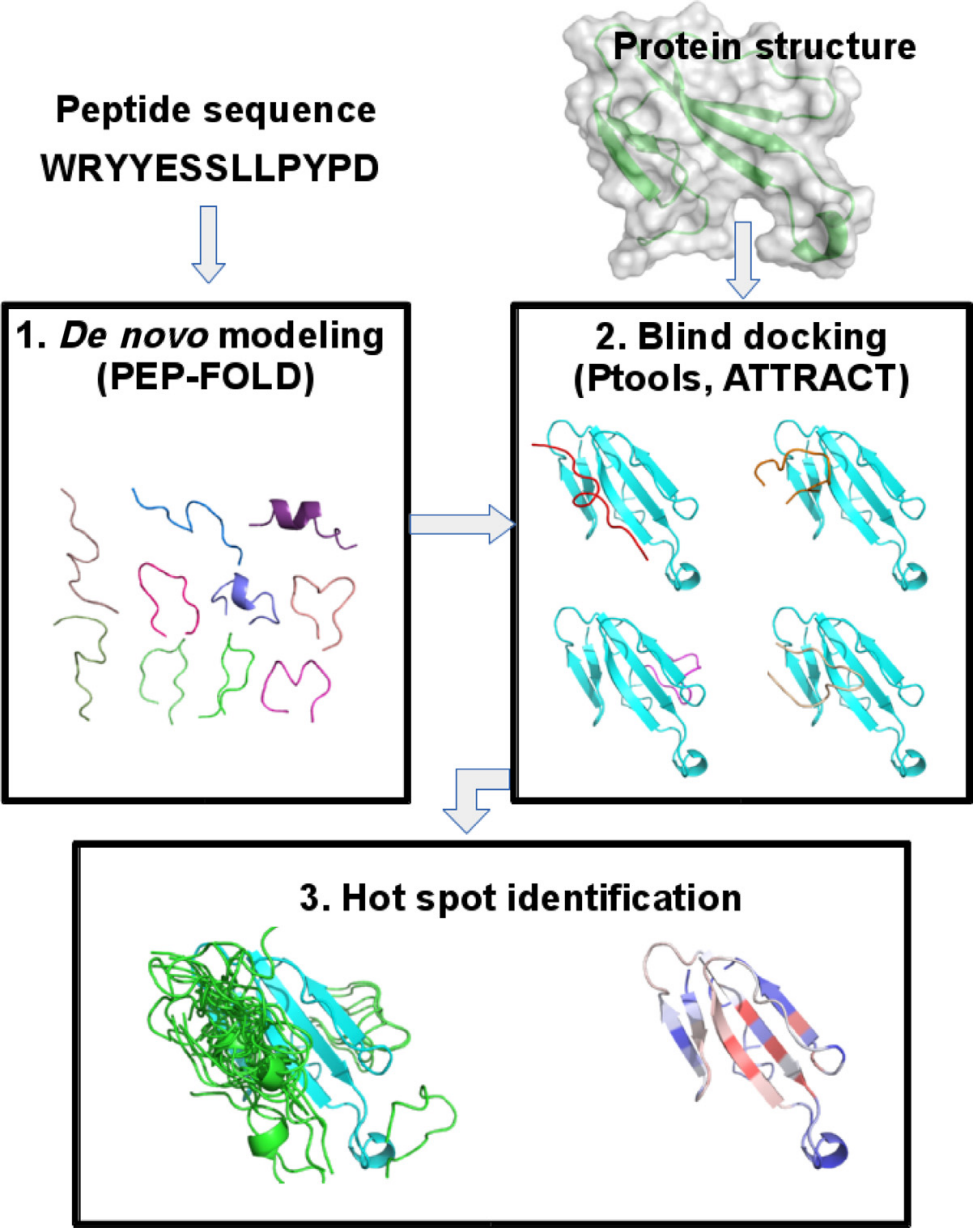
PEP-SiteFinder flowchart.

Figure 1 depicts a flowchart of PEP-SiteFinder. It consists in three main steps detailed hereafter.

#### Peptide 3D conformation generation

A first step is the prediction of an ensemble of conformations from the peptide sequence, independently of the protein. It is achieved using PEP-FOLD (25–27). PEP-FOLD relies on the concept of structural alphabets, a generalization of the concept of secondary structure extending the number of states from three (helix, strand, coil) up to 27 in our case. The states describe the conformation of fragments of four amino acids, which corresponds to the smallest peptide size PEP-SiteFinder can process. Given a peptide sequence, the probabilities of the states are predicted at each position of the peptide, and the states associated with the largest probabilities are selected. The 3D assembly is then performed from the prototype fragments associated with each of the states, using the coarse grained force field sOPEP (26). PEP-FOLD has been shown to be efficient for the *de novo* generation of peptides in solution up to 36 amino acids, which corresponds to the present upper peptide size for PEP-SiteFinder. For such sizes, the lowest energy conformations deviate, on average by 2.5 Å from the Nuclear magneticresonance (NMR) rigid cores (27).

Since peptides are known to possibly undergo conformational changes upon protein binding, it can be penalizing to consider only the lowest energy conformation. For PEP-SiteFinder, we use a modified version of PEP-FOLD that allows to sample the sub-optimal conformations. This version revisits the 3D generation procedure to return, given a peptide sequence, a diverse collection of conformations instead of searching for the lowest energy conformation. In practice, for each peptide, we generate 200 suboptimal conformations that are then clusterized. The centroids of up to the 20 clusters of lowest energy are selected for the docking step.

#### Peptide–protein blind rigid docking

For each generated peptide structure, systematic rigid docking is performed using the ATTRACT docking protocol (28) using the version 2 of the ATTRACT forcefield (29) as implemented in the PTools library (30). The ATTRACT docking protocol has been described previously (28) and only a brief description of the method is given here.

The first step of the method is the translation of the protein and the peptide into a reduced coarse-grained representation. In this second version of the ATTRACT forcefield, all atoms from the backbone are kept while side chains are represented by up to two pseudo-atoms. The energy function is the sum of two contributions, the electrostatic energy and a pairwise soft Lennard-Jones potential (29).

After this reduction step, starting points are regularly positioned around the protein, at a distance of two times the radius of the peptide from the protein surface and about 10 Å from each other. For each starting point, 260 peptide orientations are generated and an energy minimization is performed, allowing the peptide to move only in translational and rotational degrees of freedom (rigid-body docking). Since all starting positions are independent, this step is performed in parallel on our cluster on up to (arbitrarily) 180 cores, allowing us to perform a blind docking simulation usually in less than a minute for most targets. Minimized structures are then ranked by energy after merging the results from all calculating processes.

At the end of the process, up to 20 systematic rigid docking simulations have been performed. Redundant solutions are filtered out by a fast clustering procedure. Poses are ranked by energy and are picked one after another starting with the ligand with the lowest energy. If a ligand has a Root-Mean-Square Deviation (RMSD) of less than 1 Å with respect to previously found clusters this ligand is considered to be redundant and is discarded. Otherwise this ligand is considered to represent a new cluster. To keep the algorithm in O(*n*) with respect to the number of poses, only the latest 50 clusters are compared to a new ligand.

After this clustering step, the best solutions from each docking simulation are aggregated and ranked by their energy of interaction with the protein.

#### Residues at peptide–protein interface

A last step consists in assessing the propensities of protein residues to interact with the peptide. These are defined over the 50 best poses ranked according to the ATTRACT2 force field. For each pose, protein residues at the peptide interface are defined as the residues having at least one heavy atom at a distance of less than 5 Å of any peptide heavy atom. The propensity of a residue *r* is then calculated as the fraction of times it has been at the peptide–protein interface: $p_r = 100 \sum _{i=1}^{50} p_r^i /
                                50$$p_r = 100
                                \sum _{i=1}^{50} p_r^i /
                            50$ where *i* corresponds to the 50 best poses and $p_r^i$$p_r^i$ is one of (0, 1), 1 meaning the residue *r* is at peptide–protein interface for pose *i*.

### Comparison with PepSite

To compare our results with those of PepSite (21), we have submitted the complete collection of peptides to the PepSite2 web server. Results were returned for only the subset of peptides of size less than 11 amino acids. To assess the residues predicted at protein–peptide interface, we have proceeded in a similar way than for the calculation of residue propensities to be at the interface. However, since PepSite only returns one centroid per residue, and since we could not find a clear equivalence in terms of atomic position of a residue, we have, in order to keep the comparison as fair as possible, identified the protein residue contacted considering only the peptide alpha-carbons for PEP-SiteFinder and the centroids for PepSite, using a distance threshold or 6.5 and 10 Å, respectively. Only protein heavy atoms have been considered.

### Comparison with a pocket binding site identification method

We have used the fpocket (31) pocket detection software on all protein, using the apo conformations, to identify small compound binding pockets. Since fpocket has been reported to identify the pockets in interaction with small ligands at a success rate over 90% in the best three pockets, we have estimated the fraction of protein residues interacting with the ligand and belonging to the three top pockets identified.

## WEB INTERFACE

### Input

PEP-SiteFinder takes as an input a protein structure (PDB format) and a peptide sequence. The size of the input sequence must be between four and 36 amino acids (see ‘Materials and Methods’ section). There is in theory no limit about the protein size, but proteins including non polypeptidic chains (e.g. nucleic acids) are presently not accepted. Also note that the docking process discards all the hetero atoms of the input file. To test the service, the user can run a pre-configured test (GRIP1 PDZ domain in complex with liprin C-terminal peptide in interaction with the 8-mer peptide ‘ATVRTYSC’ which corresponds to the chain D of 1N7F). Even though both PEP-FOLD and rigid docking steps are rather fast, a typical run of PEP-SiteFinder requires up to 30 min and more, depending on the size of the protein, the number of peptide conformations generated using PEP-FOLD and the server load. Information about the job progress is periodically updated.

### Output

PEP-SiteFinder provides several outputs. The first consists in an interactive page allowing to browse the 3D structure of the best complexes generated, to identify the protein residues close to the different peptide poses sorted according to their ATTRACT2 scores, or the protein residues having large predicted propensities to be at peptide–protein interface. This facility depends on Jmol (32) and thus requires a Java plug-in to be installed. The user can also download the PDB files corresponding to the protein with the interaction propensities set in the temperature factor field (columns 61–66), and to the peptide poses, organized as a multiple model PDB, together with a PyMOL script to drive the off-web analysis of the results. Finally, a file listing the propensities per residue is also returned.

## RESULTS

### Example application on the PriA deoxyribonucleic acid (DNA) helicase—ssDNA-binding proteins (SSB) peptide interaction

**Figure 2.**
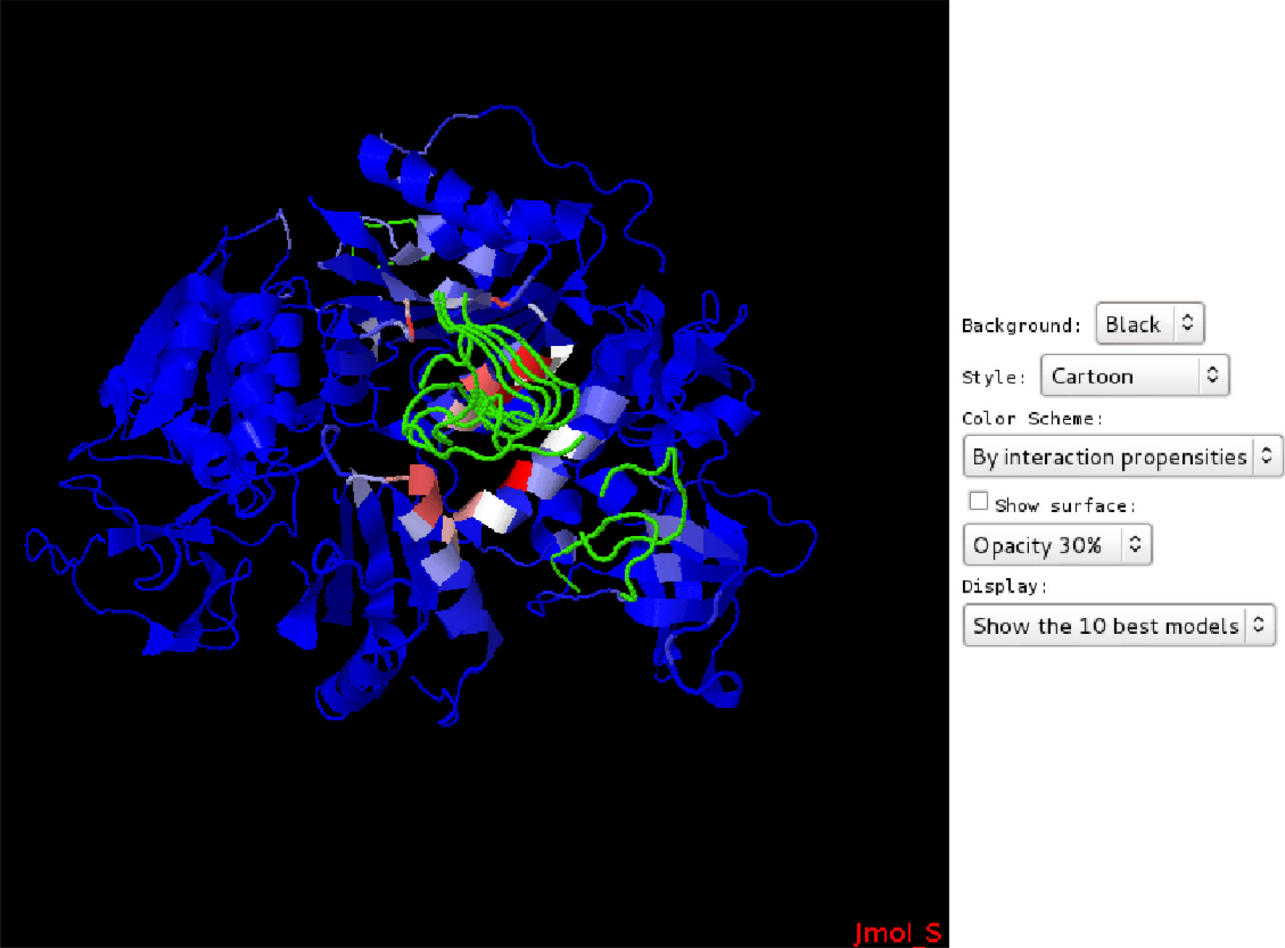
PEP-SiteFinder interactive page for the exploration of the best poses for the PriA helicase—SSB peptide complex (PDB code: 4NL8). Protein residues are colored according to their predicted propensities, from blue (0) to red (100).

We illustrate the interest of PEP-SiteFinder in the context of the Critical Assessment of Predicted Interactions (CAPRI) contest (33) target 66. Its object was the complex between the PriA DNA helicase and a SSB peptide. Before the completion of replication, collisions between cellular DNA replication machinery (replisomes) and damaged DNA or immovable protein complexes can occur and dissociate replisomes. This potentially lethal problem is resolved by the PriA DNA helicase which identifies replication forks via structure-specific DNA binding and interactions with fork-associated ssDNA-binding proteins (SSBs). The characterization of the interaction between PriA and SSBs is thus of particular interest. However, the mechanism by which PriA binds replication fork DNA and coordinates subsequent replication restart reactions have remained unclear until high resolution structural information was obtained by crystallography (34). Given the sequence of a SSB peptide and the structure of PriA DNA helicase, Figure 2 shows the 10 best poses of a SSB peptide of sequence ‘WMDFDDDIPF’ (shown in green) bound to PriA Helicase returned by PEP-SiteFinder, as could be explored in the PEP-SiteFinder result page. The colors of residue propensities to interact vary from red (large propensities—100) to blue (low propensities—0). The best spot returned corresponds, for this complex, to the actual interaction site.

### Tests on the PeptiDB dataset

**Figure 3.**
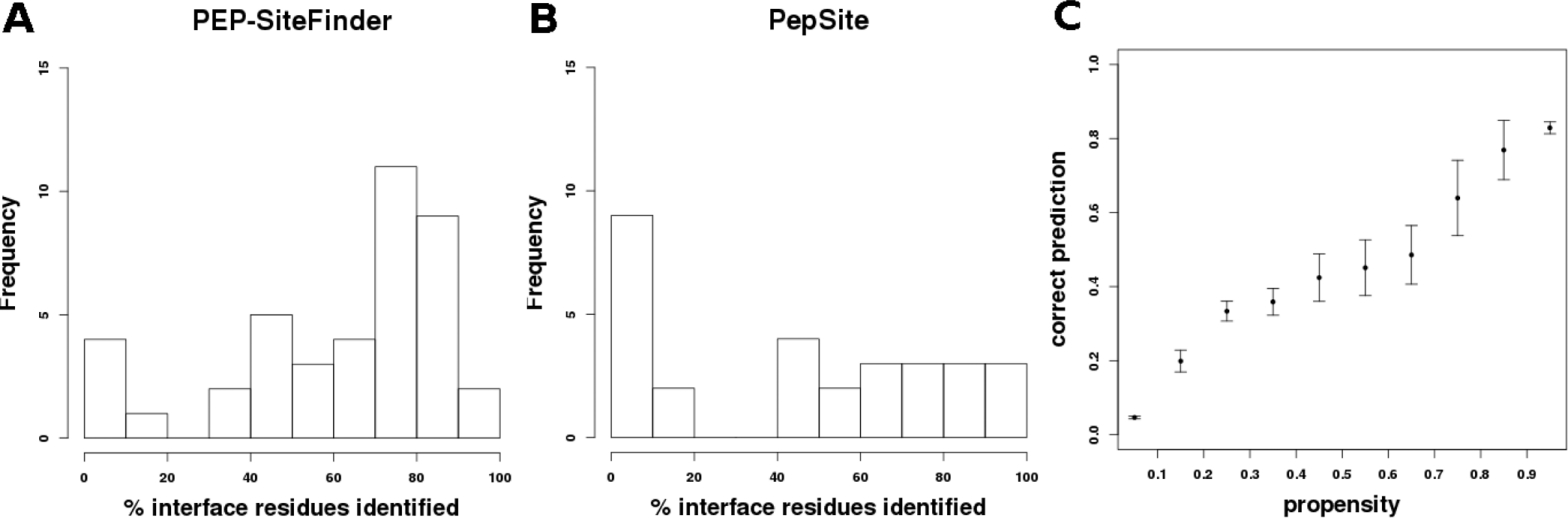
PEP-SiteFinder (**A**) and PepSite (**B**) performance over the PeptiDB core subset. Fraction of residues of the binding site contacted by the 10 best poses. (**C**) Probability that a residue is in the binding site (correct prediction) as a function of the propensity. The error bars correspond to the standard deviation estimated over five independent runs.

We have assessed the performance of PEP-SiteFinder over the complete PeptiDB collection (see Supplementary data), by searching on the surface of the unbound protein conformation the experimental peptide binding site. We first focus on the results obtained for the PeptiDB core subset of 41 complexes, and we first analyze how the 10 best poses generated by PEP-SiteFinder target the actual interaction patch (summarized Figure 3A). A major result is that the 10 best poses generated by PEP-Sitefinder fail to match any of the protein residues interacting with peptide for only four cases, i.e. for only 10% of the cases. For all other complexes, the 10 best poses generated by PEP-SiteFinder return, to different extents, relevant information about candidate residues at peptide–protein interface. Actually, piling up the analyses of the 10 best poses allows to identify >50% of the interacting residues for as much as 71% of the complexes. This strongly suggests, firstly, that the best poses, even starting with *de novo* predicted conformations can target the right protein patch and secondly, that piling up the best poses can have added value. We have also looked at the impact of the peptide conformation on the correct identification of the binding site. Overall, we find that the 20 conformations generated by PEP-FOLD approximate the conformation of the peptide in the complex at 3.5 Å RMSD, on average. The corresponding value is of 3.4 Å for the peptides of the 10 best poses and no significant deviation was observed for targets for which PEP-SiteFinder failed. This suggests that the quality of the PEP-FOLD conformations is intrinsically sufficient for the blind identification of the binding site, although the exact quantification of the minimal approximation to allow it is the matter for further investigation.

On the same data, PepSite returned results for only 29 complexes (peptide size upper limit of 10 amino acids), and could not identify any residue in the correct region for eight complexes, i.e. 27% of the cases. We also find that PepSite could identify more than 50% of the interacting residues among the 10 best poses for 48%. This highlights the added value of the PEP-SiteFinder 3D approach by comparison with a knowledge based approach such as PepSite. However, PepSite and PEP-SiteFinder both fail, for only one target suggesting added value could be found in a combination of the two approaches.

Interestingly as well, we found that the top three pockets identified by a pocket identification program such as fpocket do not correspond to the peptide binding site for 11 cases. For all these cases, PEP-SiteFinder returned relevant information. Supplementary Figure S1 depicts one such case. Despite fpocket top three pockets match to some extent the peptide binding site for four cases where PEP-SiteFinder fails, this highlights the interest of a peptide-specific approach.

Supplementary Figure 3C shows, as averaged over five independent PEP-SiteFinder runs, that large propensity values are associated with large probability values that the residues are located at the peptide–protein interface. The observed probability that a residue is actually at peptide–protein interface is of over 80% for propensity values >80%. Over all the 41 complexes, we find that the fraction of cases for which it would be possible to identify a residue at the peptide–protein interface considering the residue with the largest propensity is of 56%, increasing up to 71 and 73% considering the five and 10 best propensities, respectively. Interestingly also, increasing the distance cutoff to identify the residues contacted by the poses to 10 Å, the corresponding fractions are of 73, 76 and 78% suggesting some of the residues with the best propensities are in the vicinity of the binding site. However, we recall that presently the propensities are estimated residue per residue, i.e. not considering the proximity of the residue on the protein surface. To summarize, our results show that the combined analysis of the best scored poses and the propensities can be of great interest to identify candidate residues on the protein surface.

Finally, we briefly comment on the results obtained for PeptiDB complexes annotated as undergoing large conformational changes and for which the peptide binding site is accessible. Most often, the conformational changes correspond to the conformational modification of one or several loops upon peptide binding. Interestingly, we observe that PEP-SiteFinder is able to propose valuable predictions for all such cases. In our understanding, the diversity of the peptide conformation used by PEP-SiteFinder can accommodate such structural differences. Two such examples are depicted in the Supplementary data. These few cases also suggest that PEP-SiteFinder should be able to provide confident prediction with low quality conformations such as could be built by homology modeling, although this remains the subject for further investigation.

## CONCLUSION

PEP-SiteFinder is a tool to predict peptide-binding sites given a protein structure and a peptide sequence. Its strategy is to generate 3D conformations of the peptide from its sequence and then to use a rigid docking approach that scans the complete protein surface to extract information about the protein residues likely to be located at peptide–protein interface. Though PEP-SiteFinder relies on approximate peptide conformations, our results show that such an approach is effective, and performs, on average, better than a knowledge based approach such as PepSite. A counterpart of such strategy is that it is much more computer intensive. Nevertheless, being much slower than PepSite, it remains fast enough for a 3D approach, typical execution times being on the order of 30 min to 1 h. Several directions can be considered to improve PEP-SiteFinder, ranging from the identification of the patches of protein residues with large propensities, to revisiting the generation of the 3D conformations or enhancing complex scoring. However PEP-SiteFinder, in its present version, already provides useful information to guide mutagenesis experiments to probe peptide–protein interactions or to provide starting points for more accurate peptide–protein docking experiments.

### ACCESSION NUMBER

PDB ID: 4NL8.

### SUPPLEMENTARY DATA

Supplementary Data are available at NAR Online.

### FUNDING

French IA bioinformatics BipBip grant; INSERM UMR-S 973; Ressource Parisienne en Bioinformatique Structurale. Funding for open access charge: INSERM UMR-S 973.

*Conflict of interest statement*. None declared.

## Supplementary Material

Supplementary Data
