# Supplementary material for: PEP-SiteFinder: a tool for the blind identification of peptide binding sites on protein surfaces
Source: Nucleic Acids Res. 2014 May 6;42(Web Server issue):W221–6. doi: 10.1093/nar/gku404 (PMC4086095; doi:10.1093/nar/gku404)
Supplement: Supplementary Data [file supp_42_W1_W221__index.html]

Supplementary Data 

# PEP-SiteFinder: a tool for the blind identification of peptide binding sites on protein surfaces

## Supplementary Data

**Files in this Data Supplement:**

- Supplementary Data
