# Supplementary material for: PEP-SiteFinder: a tool for the blind identification of peptide binding sites on protein surfaces
Source: Nucleic Acids Res. 2014 May 6;42(Web Server issue):W221–6. doi: 10.1093/nar/gku404 (PMC4086095; doi:10.1093/nar/gku404)
Supplement: Supplementary Data [file supp_gku404_nar-00650-web-b-2014-File008.pdf]

Table 1. PeptiDB Peptide-protein complexes. L: protein size. p: peptide size. p s2: peptide secondary structuration (c, b, h correspond to coil, extended and helical, respectively) Res, pH: Resolution and pH at which the structure was solved. For each complex, the fraction of residues at peptide-protein interface contacted by the 10 best poses are detailed for PEP-SiteFinder (PSF), and PepSite (PSite) when possible. fpk: fraction of protein residues involved in the interaction with the peptide and identified in the 3 best pockets detected by the fpocket program. *: complexes for which the peptide binding site is not accessible in the apo conformation.

PDBid	L	p	p s2	Res	pH	PSF	PSite	fpk
peptidb core subset								
1AWR	164	6	c	1.58	8.4	100.0	68.8	84.2
1D4T	104	11	c	1.10	8.0	0.0	-	26.9
1DDV	104	6	c	1.90	8.0	44.4	0.0	0.0
1ELW	117	8	c	1.60	8.5	73.3	51.1	40.0
1ER8	330	8	c	2.00	-	76.9	41.4	82.2
1GYB	122	5	c	1.90	6.5	75.0	0.0	0.0
1HC9	81	13	b2	1.80	7.5	41.6	-	47.6
1JBU	244	15	c	2.00	7.5	73.6	-	5.9
1JWG	140	5	c	2.00	7.5	85.7	0.0	75.0
1LVM	219	6	c	1.80	8.5	0.0	0.0	12.5
1MFG	95	9	c	1.25	4.6	75.0	15.9	15.0
1NTV	152	10	c	1.50	7.5	40.0	84.4	72.4
1NVR	264	5	c	1.80	7.5	88.8	93.3	0.0
1NX1	173	11	c/h	2.00	6.2	78.5	-	0.0
1OAI	59	9	c	1.00	6.5	90.0	85.7	76.5
1OU8	106	8	c	1.60	5.7	73.6	4.8	46.4
1RXZ	245	11	c	2.00	9.0	61.1	-	17.8
1SFI	223	14	c	1.65	8.0	61.9	-	28.9
1SSH	60	11	c	1.40	7.0	87.5	-	0.0
1T4F	88	9	c/h	1.90	9.0	76.4	46.6	38.2
1U0O	227	9	c	1.95	7.5	68.7	0.0	0.0
1X2R	290	9	c	1.70	5.6	80.0	89.5	66.6
1YMT	235	10	c/h	1.20	7.5	0.0	14.7	19.0
2B9H	337	12	c	1.55	6.1	90.9	-	0.0
2C3I	266	8	c	1.90	8.5	62.5	63.1	34.6
2CCH	256	12	c	1.70	5.6	71.4	-	0.0
2DS8	41	6	c	1.60	6.5	40.0	57.8	7.1
2FGR	332	8	c	1.50	8.5	44.4	0.0	0.0
2FMF	128	13	c/h	2.00	7.5	88.8	-	73.3
2FOJ	137	7	c	1.60	8.5	83.3	64.7	15.0
2H9M	304	5	c	1.90	7.5	87.5	76.5	50.0
2HO2	33	10	c	1.33	7.4	88.8	100.0	18.2
2HPL	100	5	c	1.80	8.0	90.0	92.3	50.0
2JAM	279	6	c	1.70	-	60.0	70.5	0.0
2O02	224	14	c/h	1.50	6.5	56.6	-	4.9
2P0W	319	15	c	1.90	6.5	75.0	-	19.2
2PUY	60	10	c	1.43	6.2	56.2	50.0	15.0
2VJ0	246	8	c	1.60	6.5	20.0	0.0	0.0
2ZJD	121	10	c	1.56	7.5	50.0	0.0	35.7
3D1E	366	6	c	1.90	6.2	50.0	50.0	72.0
3D9T	95	6	c	1.50	-	0.0	77.4	20.0
complexes undergoing large conformational changes								
1H6W *	151	10	c/b	1.90	5.6	31.7	0.0	16.6
1KL3	120	6	c/h	1.70	6.9	52.9	0.0	80.0
1VZQ *	250	6	c	1.54	7.4	0.0	9.0	20.0
2B1Z *	235	9	c/h	1.78	8.5	20.0	41.9	44.4
2BBA	185	14	c/h	1.65	7.8	59.2	-	0.0
2P1K	89	16	c/b	2.00	5.5	17.6	-	34.6
2P1T	211	10	c/h	1.80	8.5	44.4	64.5	0.0
2QOS	173	11	b2	1.81	8.0	85.0	-	48.4
2R7G	337	10	c/h	1.67	6.0	90.0	0.0	10.0
3BFQ *	132	15	c/b	1.34	-	13.7	-	10.9
3BU3	294	14	c/h/b	1.65	7.5	34.7	-	21.0

Table 2. Complexes structurally redundant with PeptiDB core subset. The legend is similar to that of Table 1. Equiv. reports the PeptiDB core equivalent target, based on the CATH structural classification, when possible.

PDBid / Equiv.	L	p	p s2	Res	pH	PSF	PSite	fpk
1CKA / 1SSH	57	9	c	1.50	-	62.5	0.0	75.0
1CZY / -	168	7	c/b	2.00	6.0	0.0	0.0	0.0
1DKX / 1U00	215	7	c/b	2.00	-	92.8	29.2	78.5
1EG4 / -	260	13	c	2.00	7.0	58.3	-	0.0
1IHJ / 1MFG	94	5	c	1.80	8.5	83.3	2.7	93.7
1JD5 / 3D9T	105	8	c/b	1.90	8.5	6.2	47.5	37.5
1N7F / 1MFG	86	8	c/b	1.80	5.5	88.2	10.5	57.8
1NQ7 / 1YMT	244	10	c/h	1.50	8.0	91.6	3.5	41.1
1QKZ / 2IPU	219	10	c	1.95	5.0	85.7	93.5	44.4
1SE0 / 3D9T	97	7	c/b	1.75	5.6	0.0	2.7	35.0
1T7R / 1YMT	250	10	c/h	1.40	7.4	90.9	0.0	0.0
1TP5 / 1MFG	115	6	c/b	1.54	6.9	42.8	38.8	47.8
1TW6 / 3D9T	95	6	c/b	1.71	6.5	40.0	64.5	68.7
1UJ0 / 1SSH	58	9	c	1.70	-	85.7	95.8	41.1
1W9E / 1MFG	164	5	c/b	1.56	7.5	100.0	2.7	80.9
1YUC / 1YMT	240	14	c/h	1.90	6.4	0.0	-	20.0
1YWO / 1SSH	55	10	c	1.81	7.0	100.0	95.0	64.2
2A3I / 1YMT	253	12	c/h	1.95	7.9	100.0	-	75.0
2AK5 / 1SSH	64	8	c	1.85	6.5	42.8	95.6	40.0
2D0N / 1YMT	56	9	c	1.57	6.5	57.1	82.6	35.7
2FVJ / 1SSH	258	10	c/h	1.99	8.5	90.0	0.0	5.5
2J6F / 1YMT	57	8	c	1.70	5.5	87.5	90.4	14.2
2O4J / 1SSH	240	12	c/h	1.74	7.0	90.0	-	14.2
2O9V / 1YMT	67	10	c	1.63	6.5	69.2	78.5	0.0
2P54 / 1SSH	267	12	c/h	1.79	7.0	0.0	-	66.6
3CVP / 1YMT	279	6	c	2.00	5.0	38.8	11.8	9.3

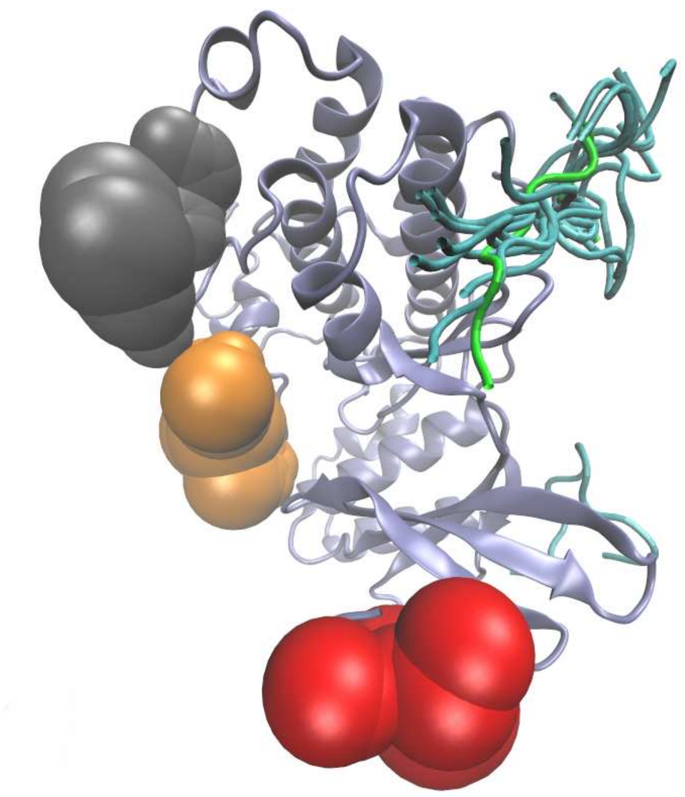

Figure 1. PEP-SiteFinder and pocket binding sites for 2B9H predicted by fpocket. The three best pockets are depicted using large spheres, with a different color for each pocket. green: peptide experimental conformation. cyan: PEP-SiteFinder best poses.

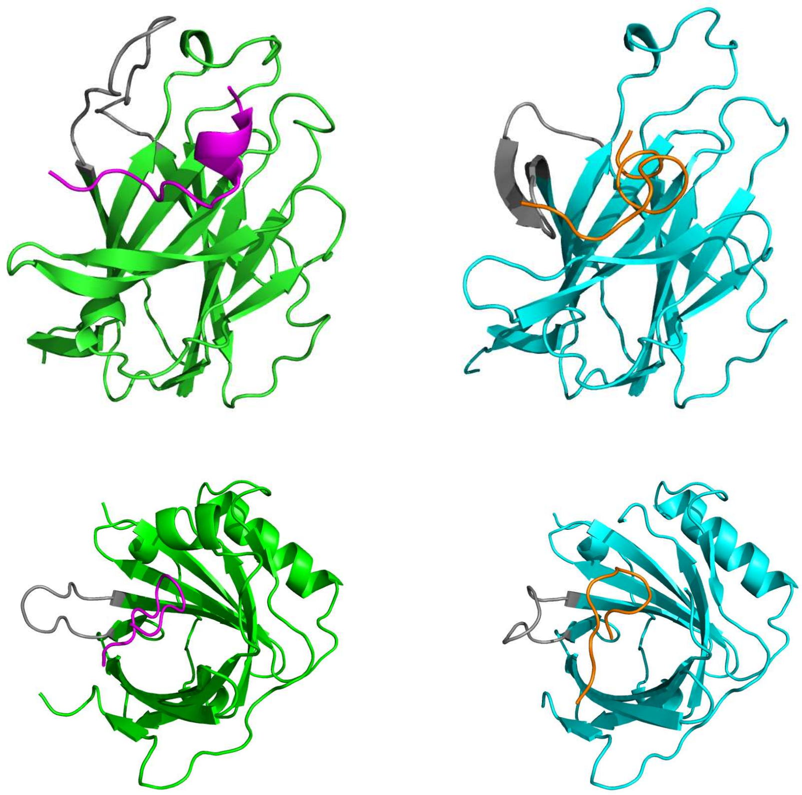

Figure 2. PEP-SiteFinder best pose for 2 complexes with conformational change upon binding. Top: 2BBA, bottom: 2QOS. Left: holo conformation. Right: PEP-SiteFinder rank 1 pose on the protein apo conformation.
